# Polyfunctional Specific Response to Echinococcus Granulosus Associates to the Biological Activity of the Cysts

**DOI:** 10.1371/journal.pntd.0004209

**Published:** 2015-11-17

**Authors:** Linda Petrone, Valentina Vanini, Elisa Petruccioli, Giuseppe Maria Ettorre, Vincenzo Schininà, Elisa Busi Rizzi, Alessandra Ludovisi, Angela Corpolongo, Giuseppe Ippolito, Edoardo Pozio, Antonella Teggi, Delia Goletti

**Affiliations:** 1 Translational Research Unit Department of Epidemiology and Preclinical Research, "L. Spallanzani" National Institute for Infectious Diseases (INMI), Rome, Italy; 2 Unit of Surgery and Transplantation "Interaziendale" Department, P.O.I.T., Polo Ospedaliero Interaziendale San Camillo-INMI Lazzaro Spallanzani, Rome, Italy; 3 Department of Radiology, "L. Spallanzani" National Institute for Infectious Diseases (INMI), IRCCS, Rome, Italy; 4 Department of Infectious, Parasitic and Immunomediated Diseases, Istituto Superiore di Sanità (ISS), IRCCS, Rome, Italy; 5 Clinical Department, National Institute for Infectious Diseases (INMI), IRCCS, Rome, Italy; 6 Scientific Direction, National Institute for Infectious Diseases (INMI), IRCCS, Rome, Italy; 7 Department of Infectious and Tropical Diseases, Sant'Andrea Hospital, "Sapienza" University, Rome, Italy; Hitit University, Faculty of Medicine, TURKEY

## Abstract

**Background:**

Cystic echinococcosis (CE) is a complex disease caused by *Echinococcus granulosus* (*E*.*granulosus*), and its immunophatogenesis is still not clearly defined. A peculiar feature of chronic CE is the coexistence of Th1 and Th2 responses. It has been suggested that Th1 cytokines are related to disease resistance, whereas Th2 cytokines are related to disease susceptibility and chronicity. The aim of this study was to evaluate, by multi-parametric flow cytometry (FACS), the presence of CE specific immune signatures.

**Methodology/Principal Findings:**

We enrolled 54 subjects with suspected CE; 42 of them had a confirmed diagnosis, whereas 12 were classified as NO-CE. Based on the ultrasonography images, CE patients were further categorized as being in "active stages" (25) and "inactive stages" (17). The ability of CD4^+^ T-cells to produce IFN-γ, IL-2, TNF-α, Th2 cytokines or IL-10 was assessed by FACS on antigen-specific T-cells after overnight stimulation with Antigen B (AgB) of *E*.*granulosus*. Cytokine profiles were evaluated in all the enrolled subjects. The results show that none of the NO-CE subjects had a detectable AgB-specific response. Among the CE patients, the frequency and proportions of AgB-specific CD4^+^ T-cells producing IL-2^+^TNF-α^+^Th2^+^ or TNF-α^+^Th2^+^ were significantly increased in the “active stages” group compared to the “inactive stages” group. Moreover, an increased proportion of the total polyfunctional subsets, as triple-and double-functional CD4 T-cells, was found in CE patients with active disease. The response to the mitogen, used as a control stimulus to evaluate the immune competence status, was characterized by the same cytokine subsets in all the subjects enrolled, independent of CE.

**Conclusions:**

We demonstrate, for the first time to our knowledge, that polyfunctional T-cell subsets as IL-2^+^TNF-α^+^Th2^+^ triple-positive and TNF-α^+^Th2^+^ double-positive specific T-cells associate with cyst biological activity. These results contribute to increase knowledge of CE immunophatogenesis and the disease outcome in terms of control and persistence.

## Author Summary

Cystic echinococcosis (CE) is a widespread zoonosis caused by the tapeworm *Echinococcus granulosus* (*E*.*granulosus*). CE is a complex disease, and several aspects of its immunophatogenesis are still not clearly defined. An important question is how the parasite influences the quality of the host’s immune response. A peculiar feature of chronic CE is the coexistence of Th1 and Th2 responses, and Th1 cytokines are related to disease resistance, whereas Th2 cytokines are related to disease susceptibility and chronicity. In the last few years, polyfunctional T-cells have been intensively studied in viral, bacterial and parasitic diseases to better understand if they represent a marker of protective immunity or disease activity. In the present study it is shown that the polyfunctional T-cell subsets producing Th2 cytokines associate with the active stages of CE. These results suggest that the cells characterized by a superior functional capacity are linked to an increased biological cyst activity rather than to a protective role. These results may contribute to increase the knowledge of CE immunophatogenesis and the disease outcome in terms of control or persistence.

## Introduction

Cystic echinococcosis (CE) is a widespread zoonosis caused by the larval stage of the tapeworm *Echinococcus granulosus* (*E*.*granulosus*) [1].

CE is also a complex disease, and several aspects, such as its natural history, parasite-host interplay, poor response to treatment, and predisposition to persistence are still not clearly defined.

An important question is how the parasite may influence the quality of the host’s immune response. A peculiar feature of chronic CE is the coexistence of Th1 and Th2 responses. It has been suggested that Th1 cytokines are related to disease resistance and in contrast, Th2 cytokines are associated with disease susceptibility and chronicity [2]; high levels of Th1 cytokines are found in patients who were successfully responding to treatment, whereas high levels of IL-4 and IL-10 occur in patients who did not [3–5]. This result indicates that the IL-10/IL-4 endogenous production induced by CE may impair Th1 response, allowing for *E*. *granulosus* persistence [6].

The nature and amount of antigens released by the parasite may play key roles in these immunoregulation mechanisms. For instance, *E*. *granulosus* Antigen B (AgB), one of the most abundant antigens in the hydatid cyst fluid, modulates the host’s response, inhibiting neutrophil recruitment [7, 8] and altering dendritic cell maturation to prime T lymphocytes into a non-protective Th2 response [9]. Notably, AgB skewed Th1/Th2 cytokine ratios towards a preferentially Th2 polarization, mainly in patients with active stages [8, 10, 11].

However, despite the high number of studies on the immune response induced by *E*.*granulosus* antigens, a comprehensive analysis of the ability of AgB-specific T-cells to co-express multiple functions has not yet been performed. Better understanding of the induction of multifunctional T-cells in the human disease may help to clarify the disease outcome, as also shown in other diseases such as HIV and TB [12–16]. This could facilitate the development of new diagnostic tools and/or the clinical management of CE patients.

Therefore, the aim of this study was to simultaneously characterize the *E*. *granulosus*-specific immune response in terms of cytokine production by flow cytometry in peripheral blood mononuclear cells (PBMC) derived from prospectively enrolled CE patients with active and inactive disease after *in vitro* stimulation with AgB.

## Materials and Methods

### Study population

Patients admitted to the “L. Spallanzani” National Institute for Infectious Diseases (INMI) and Sant’Andrea Hospital with suspected CE [risk factors for CE at the interview (Table 1) and the presence of abdominal or lung cysts at the time of the visit or in the past] were evaluated for enrollment.

**Table 1:** Survey performed to enroll patients with suspected CE.

Risk factors associated to CE
Permanence in CE endemic areas (based on WHO report)
Occupational history (shepherd, farmer, butcher, etc.)
Farm-related activities
Contact with dogs that have contacts with sheep from CE endemic areas
Intake of food/water potentially contaminated by faeces from parasitized dogs
Contact with soil potentially contaminated by faeces from parasitized dogs
History of CE cases within family members

**Footnote:** CE: Cystic Echinococcosis

CE was diagnosed based on the characteristics of images [ultraonography (US), nuclear magnetic resonance or both], and serology as a confirmatory test. Information regarding demographic data, risk factors for CE, laboratory data, symptoms, treatment and cyst description were collected. Hydatid cysts were staged according to the WHO classification [17].

**Fig 1:**
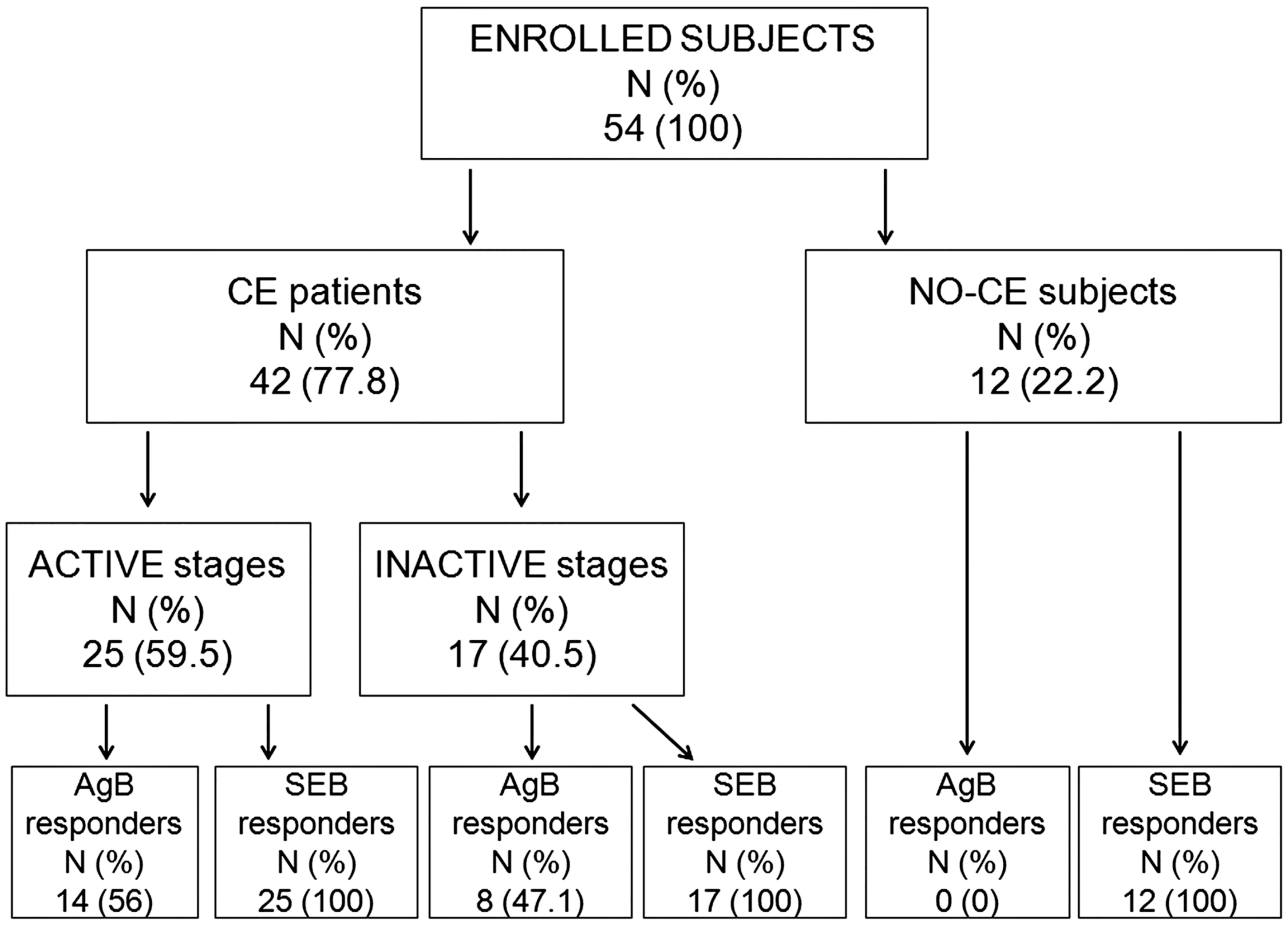
Flow chart of the enrolled subjects.

Patients having multiple cysts were classified according to the more active stage [18]. The CE patients with active (CE1 and CE2) and transitional (CE3a and CE3b) cysts were considered as a whole group because no significant differences were found in terms of the IL-4 specific immune response detected in whole blood (p = 0.1) [11]. Patients were then further classified into the categories of “active stages” (active and transitional cysts) and “inactive stages” (inactive cysts) (Fig 1).

### Ethics statement

The study was approved by the Ethical Committees of INMI (parere 34/2010; parere 28/2014) and the Sant’Andrea Hospital (Prot. C.E. n. 436/11), and all enrolled individuals provided written informed consent.

### Stimuli and antibodies

The following stimuli were used for PBMC stimulation: native AgB at 10 ug/mL, (produced at the Istituto Superiore di Sanità, as previously reported in [9]), costimulatory molecules anti-CD28 and anti-CD49d monoclonal antibodies (mAb) at 1ug/mL each (BD Bioscence, San Jose, USA), staphylococcal enterotoxin B (SEB) at 200 ng/mL (Sigma, St. Louis, MO, USA).

The fluorescently conjugated mAb used in this study were: AQUA DYE- AmCyan (Invitrogen Life Technology, Monza, IT), anti-CD4 peridinin chlorophyllprotein (PerCP)-Cy5.5-conjugated (Miltenyi Biotec S.r.l., BO, Italy), anti-CD3 allophycocyanin (APC)H7-conjugated (Miltenyi), anti-TNF-α phycoerythrin (PE)-Cy7-conjugated (eBiosceience, San Diego, CA, USA), anti- IFN-γ Horizon V450-conjugated (BD Biosciences), anti-IL-2 fluorescein isothiocyanate (FITC)-conjugated (BD Biosciences), anti-IL-4 PE (BD Biosciences), anti-IL-5 PE (Biolegend, San Diego, CA, USA), anti-IL-13 PE (Biolegend), anti-IL-10 allophycocyanin (APC)-conjugated (BD Biosciences).

### Blood processing and intracellular staining (ICS) assay

Heparinized WB was collected and processed within 2 hours. PBMC were isolated by standard methods on Ficoll-Paque Plus (GE Healthcare Bio-Sciences AB, Uppsala, Sweden) and incubated with stimuli at 37°C. Brefeldin at 10ug/mL (Sigma) was added after 1 h or 20 h of stimulation. ICS was performed after 24 h of incubation. Unstimulated cells were used as a negative control. PBMC were stained for vitality and then fixed in 2% paraformaldehyde. Therefore, the cells were resuspended in the PBS-2% FCS-0.5% saponin-2mM EDTA-1% FcR- binding inhibitor (eBioscience) buffer and stained with mAbs for surface markers and intracellular cytokines. At least 300,000 events were acquired using a FACSCanto II flow cytometer (BD Biosciences).

### Flow cytometry data analysis

Multiple-parameter flow cytometry data were analyzed using FlowJo (Tree Star Inc., San Carlos, CA) and SPICE software (provided by Dr. Roederer, Vaccine Research Center, NIAID, NIH, USA30). Cells were gated according to forward and side scatter plots and the frequency of single, double, triple, quadruple and quintuple cytokines producing CD4^+^ T-cells was evaluated using boolean combination gates. As the anti-IL-4, anti-IL-5, anti-IL-13 mAbs were conjugated with the same fluorochrome, we evaluated these cytokines as a whole, identifying them as “Th2 cytokines”. After subtracting the background values, the total cytokine production and the different cytokine subsets were expressed as frequency or percentages (proportions) of the total cytokine response. The positive CD4^+^ T-cell response was defined as the production of any cytokines (IFN-γ and/or IL-2 and/or TNF-α and/or Th2 cytokines and/or IL-10), with 0.03% as the detection limit corresponding to at least 30 analyzed events. Functional characterization of the cytokine-producing subsets was performed only in subjects with a positive AgB cytokine response. The FACS results were generated by LP and blindly re-evaluated by a co-author, EP. The agreement of the results was high (k = 0.9) and the discrepancies were solved by discussion.

### Statistical analysis

Data were analyzed using SPSS v.20 for Windows (SPSS Italia SRL, Bologna, Italy) and Prism 6 software (Graphpad Software 6.0, San Diego, CA, USA). Medians and interquartile ranges (IQR) were calculated for continuous measures; chi square for dichotomous measures. The Kruskal-Wallis test and Mann-Whitney U test were used for comparisons among several groups or pairwise comparisons, respectively. Bonferroni correction was used if needed. P values as ≤0.05 or as ≤0.016 after the Bonferroni correction were considered significant.

## Results

### Demographic and clinical characteristics of the study population

Between April 2013 and May 2015 we prospectively enrolled 54 subjects (Fig 1). Among them, 42 (77.8%) had a confirmed CE diagnosis whereas 12 (22.2%) were classified as “NO-CE subjects”, having cysts that were not related to CE. Based on the cyst stage activity, the CE patients were further classified into “active stages” [25 (59.5%)] or “inactive stages” [17 (40.5)] groups.

Demographic and clinical features are shown in Table 2. CE patients were mainly Italian, coming from the central regions [25 (73.5%)]. Serology was scored positive in 31 patients (73.8%). The 11 subjects who scored negative were characterized by a US, showing mainly inactive cysts (CE4 and CE5) [6 (54.5%)]. Seventeen (40.5%) CE patients were treated with albendazole (ABZ) prior to inclusion in the study, whereas 16 patients (38.1%) were going to start ABZ after blood collection. More than 40% of the evaluated cysts were small (diameter <5 cm), with a hepatic localization. Among the enrolled patients, only 6 (14.3%) had a farming-related job, although the majority of them [35 (83.3%)] reported risk factors, such as contact with shepherd dogs. Twenty-five (59.5%) CE patients reported symptoms, with abdominal discomfort being the most common symptom recorded.

**Table 2:** Demographical and clinical characteristics of the enrolled subjects.

	CE patients	NO-CE subjects
**N (%)**	42 (100.0)	12 (100.0)
**Median Age in years (IQR)**	63 (44–72)	60 (49–72)
**Female gender N (%)**	19 (45.2)	7 (58.3)
**Origin N (%)**		
**Italy**	34 (80.9)	12 (100.0)
**Eastern Europe**	7 (16.7)	-
**Asia**	1 (2.4)	-
**Positive serology results N (%)**	31 (73.8)	1 (8.3)
**Positive US exams N (%)**	42 (100)	0 (0)
**Previous Treatment N (%)**	17 (40.5)	1 (8.3)
**Present Treatment N (%)**	16 (38.1)	1 (8.3)
**Cyst localization N (%)**		
**Liver**	36 (85.7)	7 (70.0)
**Lung**	2 (4.8)	-
**Liver and Lung**	1 (2.4)	-
**Other localization**	3 (7.1)	3 (30.0)

**Footnote**: N: Number; IQR: Interquartile Range; y: Year; US: Ultrasound.

The 12 NO-CE subjects were sex-and age-matched donors. As the CE patients, they came from Italy (Table 2), mainly from the central regions. The serology was scored negative in almost all of the subjects with the exception of 1 person.

### Characterization of the AgB-specific total response in CE patients

To evaluate the AgB-specific T-cell responses, the cytokine profiles were assessed by ICS. All patients scored positive to the mitogen (SEB).

To evaluate the AgB-specific responses, we focused our analysis on the CD4^+^ T-cells, as in the set-up experiments, performed on a limited number of CE patients, we did not detect any CD8 T-cell specific response (none of the subjects tested).

The ability of CD4^+^ T-cells to produce IFN-γ, IL-2, TNF-α, Th2 cytokines or IL-10 was assessed in all the enrolled subjects, however, none of the NO-CE subjects had a detectable AgB-specific response.

**Fig 2:**
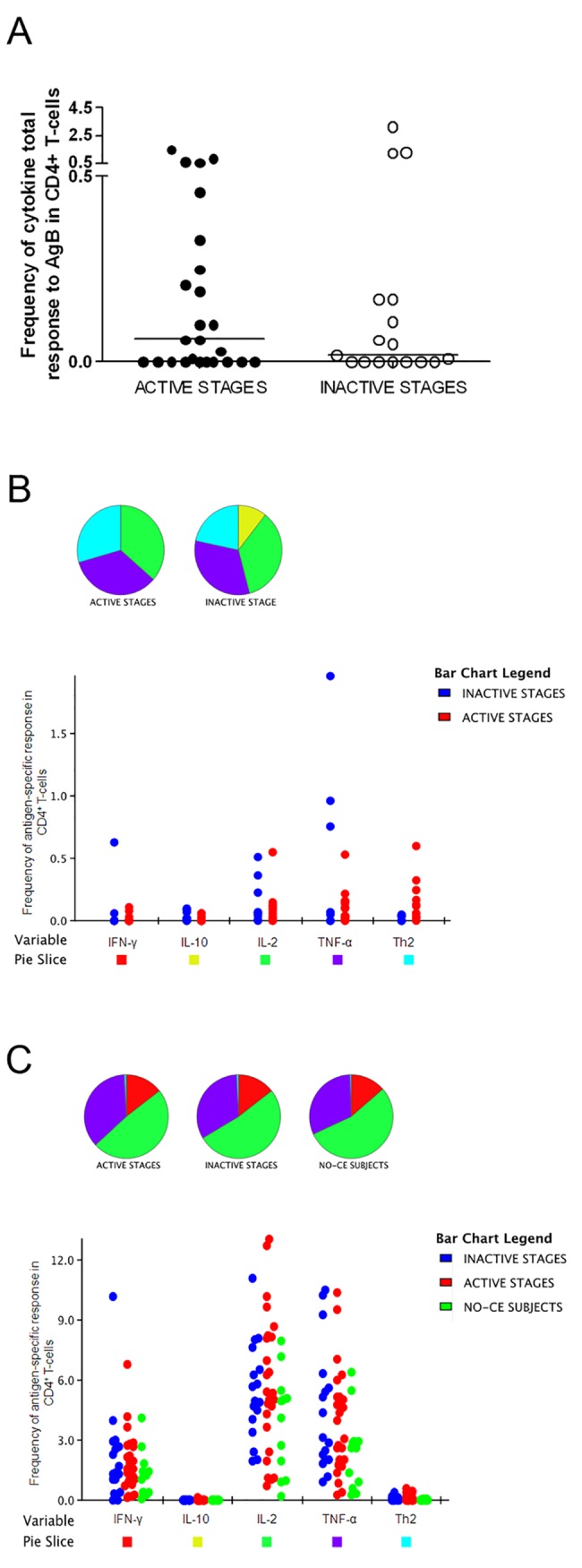
Magnitude and cytokine profile of the total AgB-specific-response. Flow cytometry evaluation of CD4^+^ T-cell response to AgB. **A.** Magnitude of the cytokine response to AgB (considering the production of any cytokine) in the “active stages” and “inactive stages” groups. **B.** Frequency of the "total cytokine response" elicited by the AgB in the “active stages” and “inactive stages” groups. **C.** Frequency of the "total cytokine response" elicited by the control stimulus SEB in the “active stages”, “inactive stages” and NO-CE subjects groups. The positive CD4^+^ T-cell response was defined as the production of any cytokines (IFN-γ and/or IL-2 and/or TNF-α and/or Th2 cytokines and/or IL-10) with 0.03% as the detection limit corresponding to at least 30 analyzed events. The horizontal lines represent the median. Black dots indicate the “active stages” CE patients, white dots indicate the “inactive stages” CE patients. Statistical analysis was performed using the Mann-Whitney test, and p value was considered significant if ≤0.05.

Among the CE patients, the magnitude of the cytokine response to AgB (considering the production of any cytokine) was higher in the “active stages” group (median: 0.06, IQR: 0–0.3) compared to the CE patients included in the “inactive stages” group (median: 0.02; IQR: 0–0.2), although it was not significant (p = 0.8) (Fig 2A). In addition, the proportion of responders to AgB was also higher in the “active stages” group than in the “inactive stages” patients (56% vs 47.1%); however, the difference was not significant (Fig 1).

We investigated the AgB-specific CD4^+^ T-cells in terms of IFN-γ, IL-2, TNF-α, IL-10 and Th2 cytokines frequency independently of the simultaneous production of the cytokines (Fig 2B). The total response in the active stages group is characterized by the production of IL-2, TNF-α and Th2 cytokines, whereas in the “inactive stages” group it is characterized by the production of IL-10 in addition to IL-2, TNF-α and Th2 cytokines. However, no significant differences were found for any of the comparisons performed.

To better define the specificity of the results obtained, we compared the AgB cytokine response with that elicited by the positive control SEB (Fig 2C). The “total cytokine response” to SEB was mostly characterized by IFN-γ, IL-2 and TNF-α cytokines in all evaluated subjects. To note, both the CE patients and the NO-CE subjects had the same cytokine profile in response to the SEB antigen.

### Polyfunctional CD4^+^ T-cells associate with cyst biological activity

A boolean gating analysis was then performed to categorize cytokine-positive cells into 31 different subsets consisting of quintuple, quadruple, triple, double or single cytokine-expressing populations.

**Fig 3:**
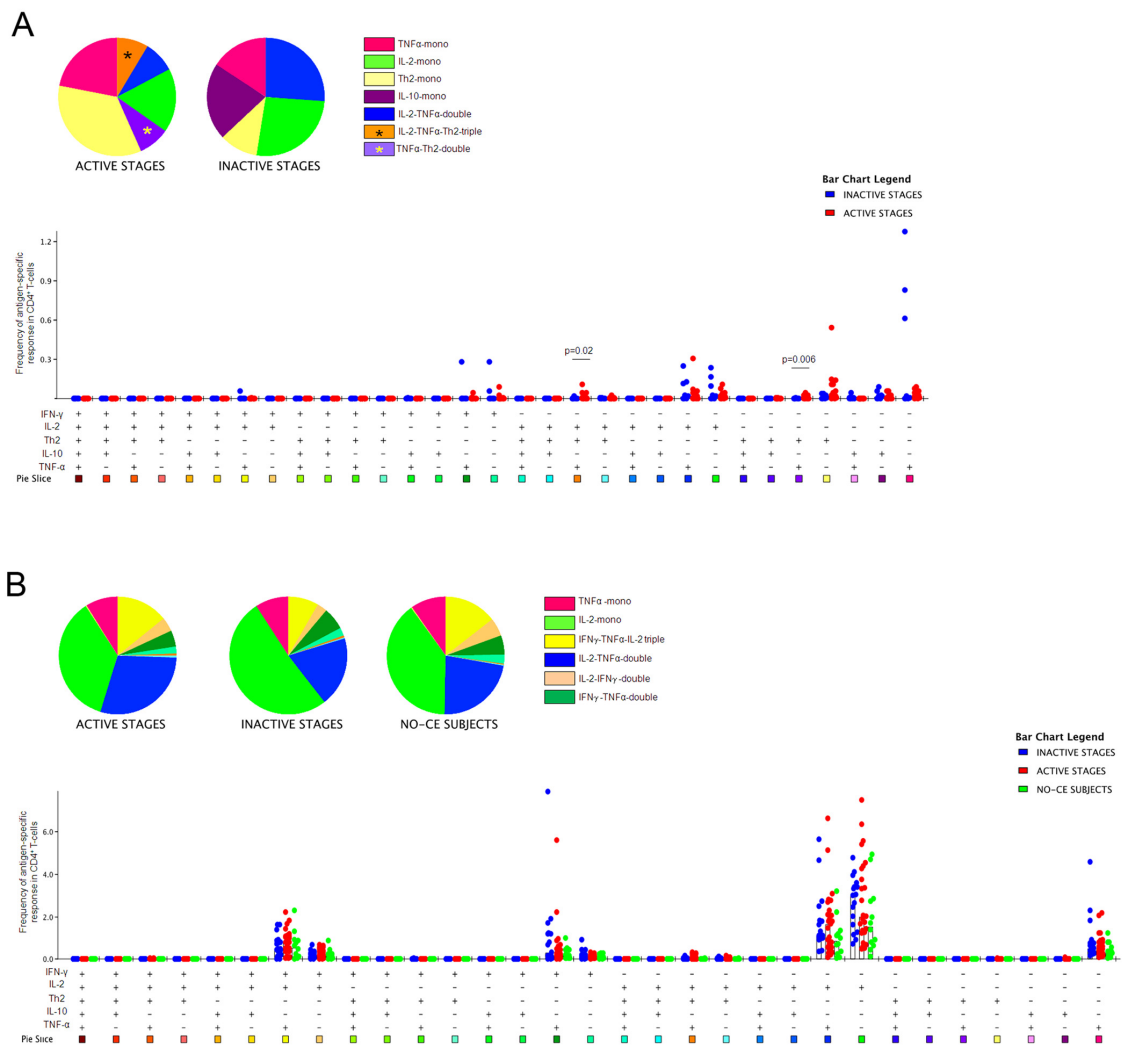
The frequency of AgB-specific CD4^+^ T-cells producing IL-2^+^TNF-α^+^Th2^+^ (triple- positive) or TNF-α^+^Th2^+^ (double-positive) was increased in the “active stages” group compared to the “inactive stages” group. Frequency of the cytokine-producing subsets of the antigen-specific response in the different groups. **A.** Cytokine profile of the AgB-specific response in the “active stages” and “inactive stages” groups. **B.** Cytokine profile elicited by the control stimulus SEB in the “active stages”, “inactive stages”, and NO-CE subjects groups. The horizontal lines represent the median; statistical analysis was performed using the Mann-Whitney test, and p value was considered significant if ≤0.05.

**Fig 4:**
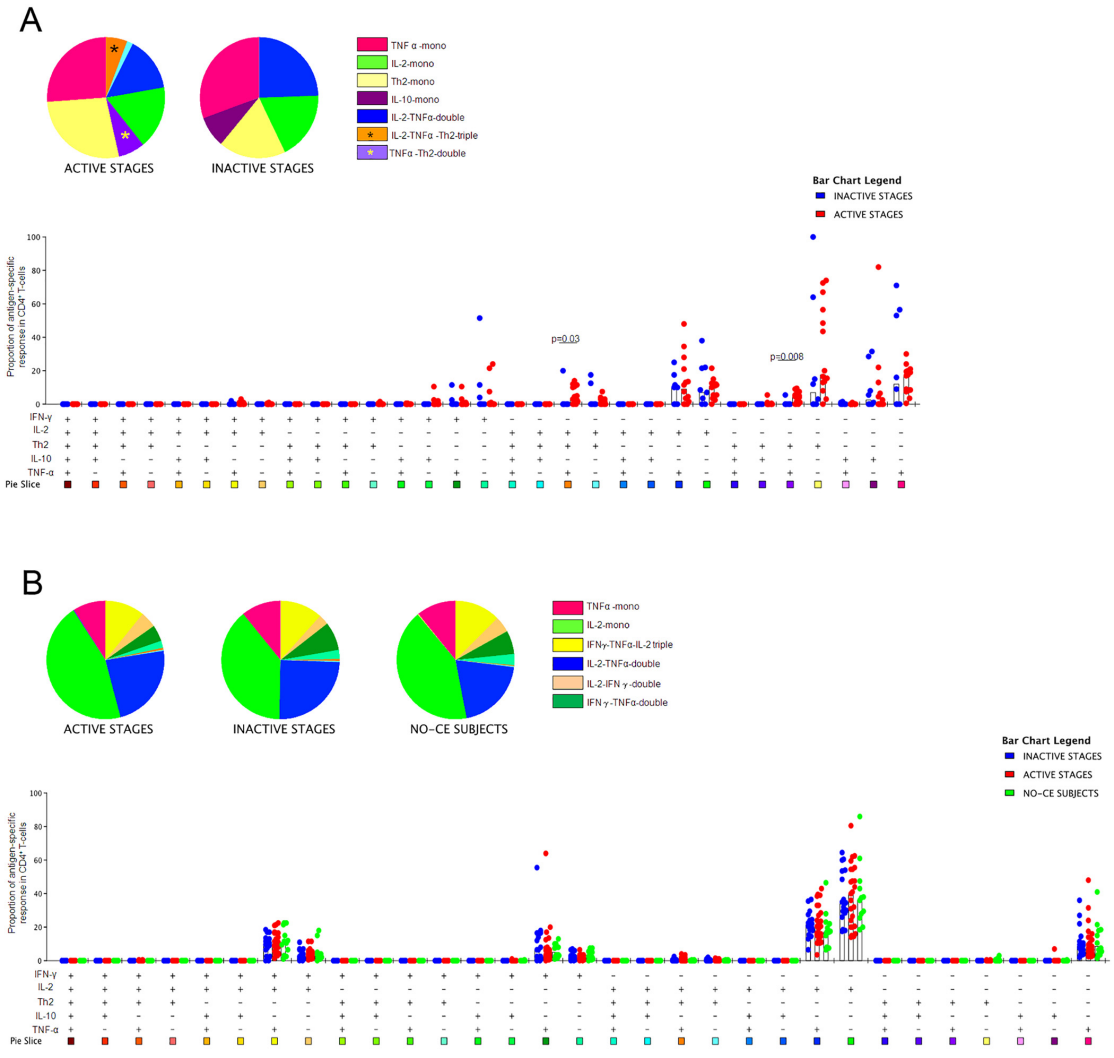
The proportion of AgB-specific CD4^+^ T-cells producing IL-2^+^TNF-α^+^Th2^+^ (triple- positive) or TNF-α^+^Th2^+^ (double-positive) was increased in the “active stages” group compared to the “inactive stages” group. Proportion of the cytokine-producing subsets of the antigen-specific response in the different groups. **A.** Cytokine profile of the AgB-specific response in the “active stages” and “inactive stages” groups. **B.** Cytokine profile elicited by the control stimulus SEB in the “active stages”, “inactive stages”, and NO-CE subjects groups. The horizontal lines represent the median; statistical analysis was performed using the Mann-Whitney test, and p value was considered significant if ≤0.05.

The frequency of AgB-specific CD4^+^ T-cells characterized to be IL-2^+^TNF-α^+^Th2^+^ (triple- positive) or TNF-α^+^Th2^+^ (double-positive) was increased in the “active stages” group compared to the “inactive stages” group (p = 0.02 and p = 0.006, respectively) (Fig 3A). Similar results were found when the proportions of the AgB-specific response were analyzed (p = 0.03 and p = 0.008, respectively) (Fig 4A). Moreover, the monofunctional CD4^+^ subset producing Th2 cytokines was increased in the “active stages” group compared to the “inactive stages” group, although the difference was not significant (Figs 3A and 4A). In contrast, the “inactive stages" group showed a higher frequency of the IL-10 monofunctional CD4^+^ T-cells subset compared to the “active stages” group. However, the difference was not significant (Fig 3A).

To better define the specificity of the result obtained, we compared the functional profile of the AgB cytokine response with that elicited by SEB. The SEB response, evaluated as frequency or proportion, was mostly characterized by the same cytokine subsets in all the subjects enrolled, independent of the CE status (Figs 3B and 4B). No significant differences were found for any of the comparisons performed in response to SEB among the two groups analyzed.

All these data suggest that only the response to *E*.*granulosus* antigens as AgB-specific triple functional IL-2^+^TNF-α^+^Th2^+^ cytokines and double functional TNF-α^+^Th2^+^ cytokines CD4^+^ T-cells associated with cyst biological activity.

### Triple-and double-functional CD4 T-cells increase in active disease

Finally, we evaluated if the proportion of the monofunctional and polyfunctional subsets are differently represented in the two groups of CE patients evaluated.

**Fig 5:**
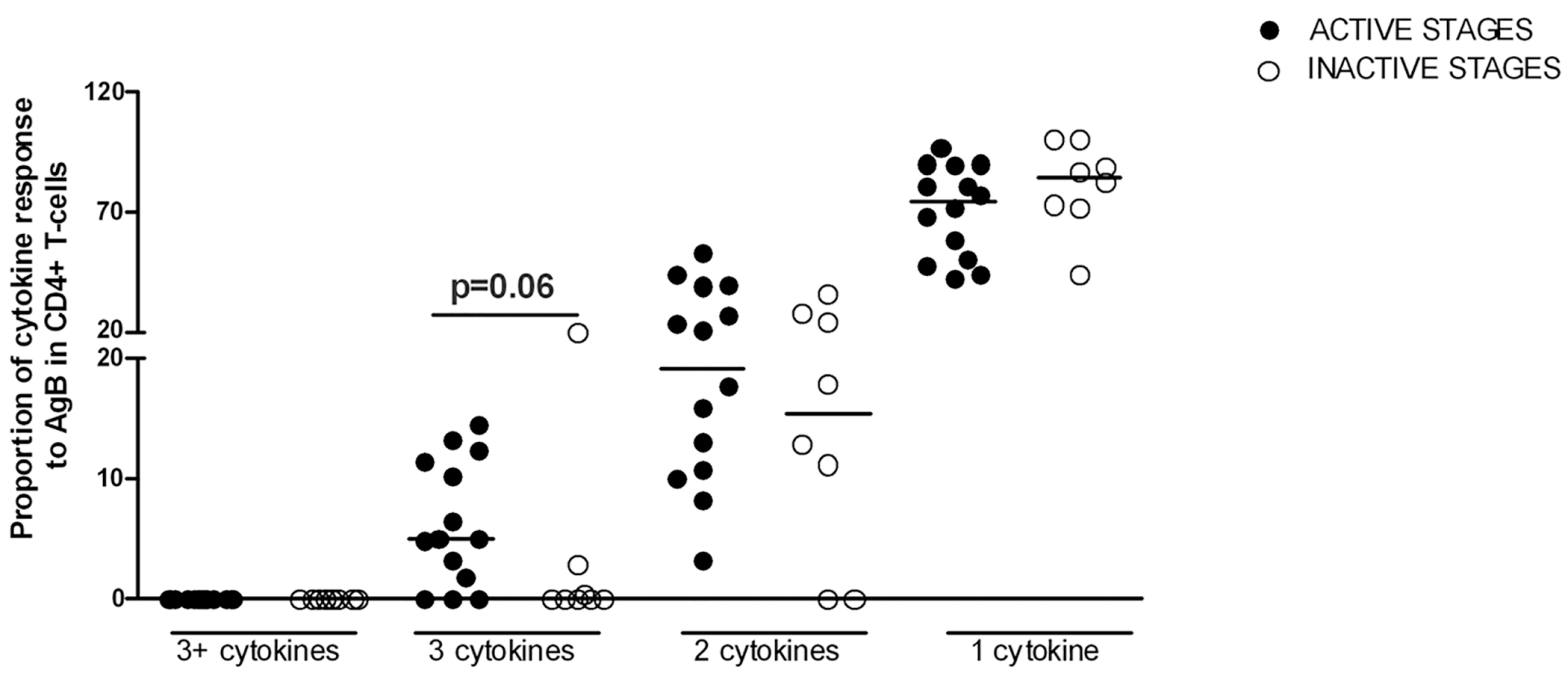
The proportion of triple-positive and double-positive T-cell subsets was increased in the “active stages” group compared to the “inactive stages” group. Proportion of the monofunctional and polyfunctional subsets in the “active stages” and “inactive stages” groups. The horizontal lines represent the median. Black dots indicate the “active stages” CE patients, white dots indicate the “inactive stages” CE patients. Statistical analysis was performed using the Mann Whitney test, and p value was considered significant if ≤0.05.

A trend for higher proportions of cells exerting 2 or 3 functions was found in the “active cysts” group compared to the “inactive cysts” group (Fig 5), supporting the previous data. In contrast, the proportion of the monofunctional subsets was similar between the two groups (Fig 5).

## Discussion

In this prospective study, we characterize, for the first time to our knowledge, the specific immune response to AgB of *E*.*granulosus* in patients with the active and inactive clinical forms of CE. We demonstrate that IL-2^+^TNF-α^+^Th2^+^ triple-positive AgB-specific CD4^+^ T-cells and TNF-α^+^Th2^+^ double-positive AgB-specific CD4^+^ T-cells associate with cyst biological activity, being significantly increased in the active CE stages. We also found an increased (but not significant) proportion of the total polyfunctional subsets in CE patients with active disease. Altogether, these results help to contribute to the knowledge of the CE immunophatogenesis, as well as the mechanisms associated with disease control and persistence. Moreover, comprehension of the pathways involved in host protection or parasite survival could help to find biomarkers for developing new tools for CE diagnosis and/or therapy monitoring.

In the last few years, polyfunctional T-cells have been intensively studied in viral, bacterial and parasitic diseases. In chronic HIV [19–21] and hepatitis C [22] viral infections, polyfunctional T-cells have been correlated with the immune protection. In contrast, their role during bacterial chronic infections such as tuberculosis is still controversial [13–16, 23] and there is currently no consensus whether polyfunctional T-cells represent a marker of protective immunity or disease activity.

Similar to viral infections, during protozoan Th1-mediated parasitic diseases, such as Leishmaniasis or Malaria, polyfunctional T-cells have been suggested to have a role in the induction of a protective immunity [24–27]. In Chagas disease, Trypanosoma cruzi-infected children, at early stages of infection, displayed mainly double- or triple-functional CD4^+^ T-cells whereas chronically infected adults showed monofunctional T-cell specific-responses [28]. In agreement with these findings, in the present study we found that the polyfunctional T-cell subsets producing Th2 cytokines associate with the active stages of CE. These results suggest that the cells characterized by a superior functional capacity are linked to an increased biological cyst activity rather than to a protective role.

Regarding the Th2 monofunctional T-cell subset, both the frequency and proportion were found increased in CE patients with active stages. These results are in agreement with the finding that active and transitional cysts are characterized by elevated IL-4 levels either in serum [29] or in AgB-stimulated blood [11]. Moreover, in patients with inactive stages, the IL-10 monofunctional T-cells subset was increased, suggesting that this cytokine has a role in parasite persistence, as previously speculated [6]. Additional studies on a larger cohort of CE patients may help to clarify if these T-cell subsets could be considered as a signature of active stages and inactive stages, respectively.

The potential limits of this study should be considered. First, we performed a cross-sectional study, analyzing a relatively small number of subjects within each group. Moreover, the small sample size hampered us from performing any intra-group analysis, evaluating each WHO CE stage. However, although a larger population size is needed to confirm these observations, the results generated here seem to be robust, as confirmed by the functional profile obtained in all the subjects enrolled, independent of CE status in response to the control stimulus SEB. In addition, the AgB cross-reactions have not been evaluated; the antigen specificity was tested in NO-CE subjects, as the prevalence of Alveolar Echinococcosis or Taeniasis is low in Italy. Finally, the analysis was restricted to those scored positive to AgB, who are not all CE patients. However, this is a limit of all the immune-based assays that measure antigen-specific responses [15, 16, 30]. The use of more antigenic molecules or peptides or different readouts or biological samples different from blood [31–34] may overcome this issue and we are currently working on this.

In conclusion, we demonstrated, for the first time, that polyfunctional T-cells subsets as IL-2^+^TNF-α^+^Th2^+^ triple-positive and TNF-α^+^Th2^+^ double-positive specific T-cells associate with biological cyst activity. Although additional studies on patients successfully responding to chemotherapy, or on patients followed over time are needed for a complete understanding of the role polyfunctional T-cells play in CE, these results may contribute to better characterizing CE immune responses and may open the door to new opportunities for generating tools for CE diagnosis and treatment monitoring.

## References

[1] Petrone L, Cuzzi G, Colace L, Ettorre GM, Busi-Rizzi E, Schinina V, et al Cystic echinococcosis in a single tertiary care center in Rome, Italy. Biomed Res Int. 2013;2013:978146 10.1155/2013/978146 24151631 PMC3789360

[2] Mezioug D, Touil-Boukoffa C. Cytokine profile in human hydatidosis: possible role in the immunosurveillance of patients infected with E.granulosus. Parasite. 2009 3;16(1):57–64. 19353953 10.1051/parasite/2009161057

[3] Rigano R, Profumo E, Di Felice G, Ortona E, Teggi A, Siracusano A. In vitro production of cytokines by peripheral blood mononuclear cells from hydatid patients. Clin Exp Immunol. 1995 3;99(3):433–9. 7882566 10.1111/j.1365-2249.1995.tb05569.x PMC1534218

[4] Rigano R, Profumo E, Ioppolo S, Notargiacomo S, Ortona E, Teggi A, et al Immunological markers indicating the effectiveness of pharmacological treatment in human hydatid disease. Clin Exp Immunol. 1995 11;102(2):281–5. 7586679 10.1111/j.1365-2249.1995.tb03778.x PMC1553412

[5] Rigano R, Profumo E, Ioppolo S, Notargiacomo S, Teggi A, Siracusano A. Serum cytokine detection in the clinical follow up of patients with cystic echinococcosis. Clin Exp Immunol. 1999 3;115(3):503–7. 10193425 10.1046/j.1365-2249.1999.00843.x PMC1905255

[6] Amri M, Mezioug D, Touil-Boukoffa C. Involvement of IL-10 and IL-4 in evasion strategies of Echinococcus granulosus to host immune response. Eur Cytokine Netw. 2009 6;20(2):63–8. 10.1684/ecn.2009.0154 19541591

[7] Shepherd JC, Aitken A, McManus DP. A protein secreted in vivo by Echinococcus granulosus inhibits elastase activity and neutrophil chemotaxis. Mol Biochem Parasitol. 1991 1;44(1):81–90. 2011156 10.1016/0166-6851(91)90223-s

[8] Rigano R, Profumo E, Bruschi F, Carulli G, Azzara A, Ioppolo S, et al Modulation of human immune response by Echinococcus granulosus antigen B and its possible role in evading host defenses. Infect Immun. 2001 1;69(1):288–96. 11119517 10.1128/IAI.69.1.288-296.2001 PMC97883

[9] Rigano R, Buttari B, Profumo E, Ortona E, Delunardo F, Margutti P, et al Echinococcus granulosus antigen B impairs human dendritic cell differentiation and polarizes immature dendritic cell maturation towards a Th2 cell response. Infect Immun. 2007 4;75(4):1667–78. 17210662 10.1128/IAI.01156-06 PMC1865679

[10] Rigano R, Buttari B, De Falco E, Profumo E, Ortona E, Margutti P, et al Echinococcus granulosus-specific T-cell lines derived from patients at various clinical stages of cystic echinococcosis. Parasite Immunol. 2004 1;26(1):45–52. 15198645 10.1111/j.0141-9838.2004.00682.x

[11] Petrone L, Vanini V, Petruccioli E, Ettorre GM, Busi Rizzi E, Schinina V, et al IL-4 specific-response in whole blood associates with human Cystic Echinococcosis and cyst activity. J Infect. 2015 3;70(3):299–306. 10.1016/j.jinf.2014.10.009 25444973

[12] Prezzemolo T, Guggino G, La Manna MP, Di Liberto D, Dieli F, Caccamo N. Functional Signatures of Human CD4 and CD8 T Cell Responses to Mycobacterium tuberculosis. Front Immunol. 2014 4 22;5:180 10.3389/fimmu.2014.00180 24795723 PMC4001014

[13] Harari A, Rozot V, Enders FB, Perreau M, Stalder JM, Nicod LP, et al Dominant TNF-alpha+ Mycobacterium tuberculosis-specific CD4+ T cell responses discriminate between latent infection and active disease. Nat Med. 2011 3;17(3):372–6. 10.1038/nm.2299 21336285 PMC6570988

[14] Day CL, Abrahams DA, Lerumo L, Janse van Rensburg E, Stone L, O'rie T, et al Functional capacity of Mycobacterium tuberculosis-specific T cell responses in humans is associated with mycobacterial load. J Immunol. 2011 9 1;187(5):2222–32. 10.4049/jimmunol.1101122 21775682 PMC3159795

[15] Petruccioli E, Petrone L, Vanini V, Sampaolesi A, Gualano G, Girardi E, et al IFNgamma/TNFalpha specific-cells and effector memory phenotype associate with active tuberculosis. J Infect. 2013 6;66(6):475–86. 10.1016/j.jinf.2013.02.004 23462597

[16] Chiacchio T, Petruccioli E, Vanini V, Cuzzi G, Pinnetti C, Sampaolesi A, et al Polyfunctional T-cells and effector memory phenotype are associated with active TB in HIV-infected patients. J Infect. 2014 6 26. 10.1016/j.jinf.2014.06.009 24975174

[17] WHO Informal Working Group. International classification of ultrasound images in cystic echinococcosis for application in clinical and field epidemiological settings. Acta Trop. 2003 2;85(2):253–61. 12606104 10.1016/s0001-706x(02)00223-1

[18] Hosch W, Junghanss T, Stojkovic M, Brunetti E, Heye T, Kauffmann GW, et al Metabolic viability assessment of cystic echinococcosis using high-field 1H MRS of cyst contents. NMR Biomed. 2008 8;21(7):734–54. 10.1002/nbm.1252 18384178

[19] Kannanganat S, Ibegbu C, Chennareddi L, Robinson HL, Amara RR. Multiple-cytokine-producing antiviral CD4 T cells are functionally superior to single-cytokine-producing cells. J Virol. 2007 8;81(16):8468–76. 17553885 10.1128/JVI.00228-07 PMC1951378

[20] Betts MR, Nason MC, West SM, De Rosa SC, Migueles SA, Abraham J, et al HIV nonprogressors preferentially maintain highly functional HIV-specific CD8+ T cells. Blood. 2006 6 15;107(12):4781–9. 16467198 10.1182/blood-2005-12-4818 PMC1895811

[21] Makedonas G, Betts MR. Living in a house of cards: re-evaluating CD8+ T-cell immune correlates against HIV. Immunol Rev. 2011 1;239(1):109–24. 10.1111/j.1600-065X.2010.00968.x 21198668 PMC3025661

[22] Ciuffreda D, Comte D, Cavassini M, Giostra E, Buhler L, Perruchoud M, et al Polyfunctional HCV-specific T-cell responses are associated with effective control of HCV replication. Eur J Immunol. 2008 10;38(10):2665–77. 10.1002/eji.200838336 18958874

[23] Caccamo N, Guggino G, Joosten SA, Gelsomino G, Di Carlo P, Titone L, et al Multifunctional CD4(+) T cells correlate with active Mycobacterium tuberculosis infection. Eur J Immunol. 2010 8;40(8):2211–20. 10.1002/eji.201040455 20540114

[24] Darrah PA, Patel DT, De Luca PM, Lindsay RW, Davey DF, Flynn BJ, et al Multifunctional TH1 cells define a correlate of vaccine-mediated protection against Leishmania major. Nat Med. 2007 7;13(7):843–50. 17558415 10.1038/nm1592

[25] Macedo AB, Sanchez-Arcila JC, Schubach AO, Mendonca SC, Marins-Dos-Santos A, de Fatima Madeira M, et al Multifunctional CD4(+) T cells in patients with American cutaneous leishmaniasis. Clin Exp Immunol. 2012 3;167(3):505–13. 10.1111/j.1365-2249.2011.04536.x 22288594 PMC3374283

[26] Roestenberg M, McCall M, Hopman J, Wiersma J, Luty AJ, van Gemert GJ, et al Protection against a malaria challenge by sporozoite inoculation. N Engl J Med. 2009 7 30;361(5):468–77. 10.1056/NEJMoa0805832 19641203

[27] Roestenberg M, Teirlinck AC, McCall MB, Teelen K, Makamdop KN, Wiersma J, et al Long-term protection against malaria after experimental sporozoite inoculation: an open-label follow-up study. Lancet. 2011 5 21;377(9779):1770–6. 10.1016/S0140-6736(11)60360-7 21514658

[28] Albareda MC, De Rissio AM, Tomas G, Serjan A, Alvarez MG, Viotti R, et al Polyfunctional T cell responses in children in early stages of chronic Trypanosoma cruzi infection contrast with monofunctional responses of long-term infected adults. PLoS Negl Trop Dis. 2013 12 12;7(12):e2575 10.1371/journal.pntd.0002575 24349591 PMC3861186

[29] Tamarozzi F, Meroni V, Genco F, Piccoli L, Tinelli C, Filice C, et al Ex vivo assessment of serum cytokines in patients with cystic echinococcosis of the liver. Parasite Immunol. 2010 Sep-Oct;32(9–10):696–700. 10.1111/j.1365-3024.2010.01236.x 20691021

[30] Adekambi T, Ibegbu CC, Cagle S, Kalokhe AS, Wang YF, Hu Y, et al Biomarkers on patient T cells diagnose active tuberculosis and monitor treatment response. J Clin Invest. 2015 5;125(5):1827–38. 10.1172/JCI77990 25822019 PMC4598074

[31] Cannas A, Calvo L, Chiacchio T, Cuzzi G, Vanini V, Lauria FN, et al IP-10 detection in urine is associated with lung diseases. BMC Infect Dis. 2010 11 22;10:333,2334-10-333. 10.1186/1471-2334-10-333 21092156 PMC2995466

[32] Petrone L, Chiacchio T, Vanini V, Petruccioli E, Cuzzi G, Di Giacomo C, et al High urine IP-10 levels associate with chronic HCV infection. J Infect. 2014 6;68(6):591–600. 10.1016/j.jinf.2014.02.008 24582930

[33] Petrone L, Cannas A, Aloi F, Nsubuga M, Sserumkuma J, Nazziwa RA, et al Blood or urine IP-10 cannot discriminate between active tuberculosis and respiratory diseases different from tuberculosis in children. Biomed Res Int. 2015. 10.1155/2015/589471 PMC4540955 26346028

[34] Chiacchio T, Petruccioli E, Vanini V, Butera O, Cuzzi G, Petrone L, et al Higher frequency of T-cell response to M. tuberculosis latency antigen Rv2628 at the site of active tuberculosis disease than in peripheral blood. PLoS One. 2011;6(11):e27539 10.1371/journal.pone.0027539 22102905 PMC3213161

